# Inducible nitric oxide synthase accelerates nonalcoholic fatty liver disease progression by regulating macrophage autophagy

**DOI:** 10.1002/iid3.1114

**Published:** 2023-12-26

**Authors:** Guiyuan Jin, Xiaoying Yao, Dong Liu, Juan Zhang, Xiaobei Zhang, Yonghong Yang, Yanzhen Bi, Hui Zhang, Guanjun Dong, Huixin Tang, Shumin Cheng, Feng Hong, Meng Si

**Affiliations:** ^1^ Medical Research Center Affiliated Hospital of Jining Medical University Jining Shandong Province China; ^2^ Institute of Immune Precision Diagnosis and Therapy and Translational Medicine Affiliated Hospital of Jining Medical University Jining Shandong Province China; ^3^ Department of Clinical Laboratory Affiliated Hospital of Jining Medical University Jining Shandong Province China; ^4^ Department of Clinical Ultrasonics Affiliated Hospital of Jining Medical University Jining Shandong Province China; ^5^ Department of Infectious Disease Qingdao Municipal Hospital Qingdao Shandong Province China; ^6^ Institute of Immunology and Molecular Medicine Jining Medical University Shandong China; ^7^ Department of Gastroenterology People's Hospital of Jia Xiang Jining Shandong Province China; ^8^ School of Foreign Languages Jining Medical University Shandong China

**Keywords:** autophagy, inducible nitric oxide synthase, macrophage, nonalcoholic fatty liver disease

## Abstract

**Background:**

Cells and tissues, such as macrophages, express inducible nitric oxide synthase (INOS) after stimulation by certain factors. INOS helps mediate the macrophage inflammatory reaction, but few studies have explored how INOS affects macrophage function in nonalcoholic fatty liver disease (NAFLD).

**Objective:**

This study investigated the role of INOS‐mediated macrophage activity in NAFLD.

**Methods:**

A high‐fat diet was used to establish an NAFLD mouse model. After 12 weeks, blood was collected for immune cell and lipid analyses, and liver tissues were collected for pathological analyses with hematoxylin and eosin and Oil Red O staining. Peritoneal macrophages were extracted in situ, cultured in Dulbecco's modified Eagle's medium, and stimulated with palmitic acid to mimic in vivo conditions for further assays. Real‐time polymerase chain reaction, western blot analysis, and immunofluorescence were used to verify the expression of target genes or proteins.

**Results:**

In the NAFLD model, INOS expression in macrophages increased, and *INOS* knockdown significantly decreased the number of macrophages. Pathological examinations confirmed that *INOS* knockdown slowed NAFLD progression and macrophage infiltration during inflammation. *INOS* knockdown also enhanced phagocytosis and lipid transport by macrophages, and increased the expression of autophagy‐related molecules in macrophages, which improved the autophagy level, promoted apoptotic cell degradation, and maintained intracellular environment homeostasis.

**Conclusions:**

These results indicate a correlation between INOS expression and macrophage function in NAFLD.

## 1 INTRODUCTION

Nonalcoholic fatty liver disease (NAFLD) is a pathological metabolic disease caused by several factors (excluding alcohol or liver injury), such as metabolism, gene expression, environmental pollution, and intestinal microorganisms. The incidence of NAFLD has increased in many developing countries,^1^ occurring in approximately 24% of adults worldwide. However, this value is often underestimated owing to detection method limitations.^2^, ^3^ NAFLD includes simple fatty liver and nonalcoholic steatohepatitis (NASH), which can progress to liver fibrosis and cirrhosis, increasing the risk of hepatocellular carcinoma. NAFLD is closely associated with various metabolic diseases, including obesity, insulin resistance, type 2 diabetes, and dyslipidemia,^4^, ^5^ and except for lifestyle interventions and weight loss, clear and effective treatments do not exist.^6^, ^7^ Therefore, exploring the pathogenesis of NAFLD may provide new insights into clinical therapies.

The pathogenesis of NAFLD remains widely debated and mainly includes the “second strike” theory, fructose induction, adipose tissue dysfunction, intestinal flora disorders, abnormal bile acid metabolism, and the accumulation or lack of metal elements.^8^ The hepatic immune microenvironment, including Kupffer cells, is key in NAFLD progression. Kupffer cells (immune cells) settle in the liver, and recruited macrophage polarization plays a key role in the pathogenesis of liver inflammation, fibrosis, and NAFLD. Hepatocyte injury may induce macrophage polarization into the classic activated macrophage M1 type, secrete proinflammatory cytokines and other substances, and recruit more monocyte macrophages to aggravate liver injury.^9^

Several factors and pathways promote macrophage activation in NAFLD. First, plasma endotoxin levels increase as intestinal microbiota changes, intestinal bacterial overgrowth, and increased intestinal permeability occur in patients with NAFLD; these events are related to the gut–liver axis.^10^ Consequently, intestinal endotoxins, such as lipopolysaccharide (LPS), enter circulation and activate Kupffer cells through toll‐like receptors (TLRs).^11^, ^12^, ^13^ Second, hepatocyte damage triggers macrophage activation, and apoptotic bodies activate Kupffer cells.^14^ Specific oxidized epitopes on apoptotic cells or cell fragments act as risk molecular patterns, which also activate macrophages through pattern recognition receptors.^15^ High histidine‐rich glycoprotein expression in patients with NASH has been associated with disease staging,^14^ and several studies have demonstrated that lipotoxic hepatocytes release extracellular vesicles containing CXC chemokine ligand 10 and tumor necrosis factor (TNF)‐related apoptosis‐inducing ligand (also known as TNF superfamily member 10), which promote macrophage recruitment and activation and NAFLD progression.^16^, ^17^, ^18^ Third, free fatty acids and leptin secreted by adipose tissue activate Kupffer cells through TLRs and leptin receptors.^19^, ^20^ Moreover, increasing evidence suggests that excess cholesterol (CHO) is a potential pathogenic cofactor of NAFLD. CHO and its metabolites activate Kupffer cells through cluster of differentiation (CD) 36 and scavenger receptor A.^21^ Finally, Kupffer cells secrete proinflammatory cytokines and chemokines, which recruit circulating neutrophils and monocytes to the liver. Monocytes differentiate into proinflammatory macrophages, which amplify liver inflammation and stimulates stellate cell transformation into activated myofibroblasts, leading to fibrosis. In conclusion, functional changes of different macrophages play important regulatory roles in NAFLD development. Therefore, this study focused on changes in immune cells and macrophage function in an NAFLD disease model to further elucidate the relevant mechanisms.

Nitric oxide synthase (NOS), generally classified as constitutive NOS (cNOS) and inducible NOS (INOS), is distributed among various cells. cNOS is sub‐classified as neural NOS and endothelial NOS (eNOS). Generally, INOS is not expressed in cells; it is primarily found in macrophages stimulated by LPS, cytokines, and other factors. Once expressed, INOS remains active and independent of the intracellular Ca^2+^ concentration. eNOS and INOS exist in the liver; specifically, eNOS exists in intrahepatic blood vessels, sinusoidal endothelial cells, and hepatocytes, and INOS exists in hepatocytes and hepatic macrophages. INOS mainly produces nitric oxide (NO) at the transcriptional level, which is a free radical gas with extremely active chemical properties with a dual oxidation and antioxidation role in the oxidative stress response of tissues and organs. NO and INOS levels are significantly associated with the occurrence and development of NAFLD. Both promote lipid peroxidation in the liver, inhibit hepatocyte protein synthesis, cause glucose metabolism disorders, promote mitochondrial damage, and accelerate hepatocyte apoptosis.^22^

Considering the importance of macrophages and the uncertainties surrounding the pathogenic mechanisms of NAFLD, this study investigated how INOS in macrophages affects NALFD, which may have clinical significance for treating this disease.

## 2 MATERIALS AND METHODS

### 2.1 Animal experiments

All mice were housed at the Center for Experimental Animals of the Medical Research Centre of the Affiliated Hospital of Jining Medical University under pathogen‐free conditions throughout the experiment. C57BL/6J male mice (18–20 g, 6–7 weeks old) were purchased from Weitonglihua Co. and housed in plastic cages lined with sawdust. The animals were kept under a 12‐h light‐dark cycle, and food and water were provided ad libitum.

The mice were randomly assigned to control or methionine‐ and choline‐deficient (MCD) diet groups (*n* = 6 per group). The control mice were fed a normal diet, and the MCD diet group was fed an MCD diet. MCD food was purchased from Medicine Professionals for Laboratory Animal Diets. The animals were fed their respective diets for 2 weeks (14 days), then all mice were euthanized under anesthesia (intraperitoneal pentobarbital injection), and blood and liver samples were collected.

In addition, we obtained mice from the Immunology Department of Basic Medical Science of Jining Medical University to create male congenic wild‐type (*INOS^+/+^*) and INOS knockout (*INOS^−/−^*) mice. Then, the *INOS^+/+^* and *INOS^−/−^* mice were fed a high‐fat diet (HFD) for 12 weeks and then euthanized with carbon dioxide. Blood samples were collected for lipid and immune cell analyses. The livers were also dissected, frozen, and fixed in 4% paraformaldehyde overnight to prepare sections and then stained with Oil Red O for observation under an optical microscope or subjected to immunofluorescence staining.

All laboratory animals were housed in regulated rooms, and all operations and experiments were conducted following the National Institutes of Health Guide for Care and Use of Laboratory Animals.

### 2.2 Biochemical analysis of lipid status and liver function

Serum total CHO, triglyceride (TG), low‐density lipoprotein‐CHO (LDL‐C), alanine aminotransferase (ALT), and aspartate aminotransferase (AST) levels were measured using commercial kits (Roche Diagnostics).

### 2.3 Histopathology

Liver tissues were fixed in 4% paraformaldehyde for 24 h, embedded in paraffin, and cut into thin slices for hematoxylin and eosin (HE) staining. In addition, 5 µm of frozen liver sections were fixed and stained with Oil Red O (Sigma‐Aldrich).

### 2.4 Flow cytometry

Cells were isolated from the blood and liver samples and then stained for CD3, CD4, CD8, F4/80, and CD11c (eBioscience) and analyzed by flow cytometry using CytoFLEX S (Beckman Coulter).

### 2.5 Cell culture

Age‐and sex‐matched *INOS* ^*−/−*^ and *INOS* ^*+/+*^ mice were injected intraperitoneally with 1 mL of 3% sterile thioglycolate broth (Sigma‐Aldrich). Four days later, the peritoneal macrophages were harvested and washed with a serum‐free medium. The cells were cultured in Dulbecco's modified Eagle's medium (Corning) supplemented with 10% fetal bovine serum (Invitrogen‐Gibco/Thermo Fisher Scientific) and 1% penicillin/streptomycin (Solarbio). Cells were seeded in 24‐well plates (Corning Inc.) at 2 × 10^5^ cells/well for immunofluorescence, in 12‐well plates (Corning Inc.) at 5 × 10^5^ cells/well for RNA extraction, or in six‐well plates (Corning Inc.) at 2 × 10^6^ cells/well for western blot (WB) analysis. All cells were maintained at 37°C in a 5% CO_2_ atmosphere.

### 2.6 Palmitic acid (PA) stimulation

Cells were treated with 0, 100, 200, or 400 μM of PA (Sigma‐Aldrich) mixed with bovine serum albumin (BSA) for 12 h or 200 μM for 0, 12, and 24 h. Then, the cells were harvested for protein detection, RNA extraction, or immunofluorescence.

### 2.7 Immunofluorescence and confocal microscopy

To investigate the changes and correlation between INOS and autophagy in macrophages, the mouse livers were dissected and frozen to prepare sections for immunofluorescence analyses. To detect autophagy changes in macrophages after *INOS* knockout, the peritoneal macrophages of *INOS^+/+^* and *INOS^−/−^* mice were stimulated with PA, and the autophagy level was detected by immunofluorescence. The treated cells were seeded on a glass slide and fixed, and the liver sections were fixed; then, both were treated with 0.1% Triton X‐100 and 10% BSA for 1 h. The samples were incubated with primary antibodies at 4°C overnight. The primary antibodies used were anti‐light chain 3B (LC3B) (Cell Signaling Technologies; 1:100 dilution), anti‐INOS (Cell Signaling Technologies; 1:100 dilution), and anti‐Mac‐3 (BD Biosciences; 1:100 dilution). After washing three times with phosphate‐buffered saline, the samples were incubated with secondary antibodies for 1 h at 37°C. The secondary antibodies used were anti‐rabbit CoraLite594 (red; Proteintech; 1:500 dilution), anti‐mouse CoraLite594 (red; Proteintech; 1:500 dilution), anti‐rabbit CoraLite488 (green; Proteintech; 1:500 dilution), and anti‐mouse CoraLite488 (green; Proteintech; 1:500 dilution). After washing, 4′,6‐diamidino‐2‐phenylindole (Beyotime Biotechnology) staining was performed for 10 min. Autophagy flow was detected using the mCherry‐egfp‐lc3b adenovirus (HanBio Therapeutics). The cells were then washed and treated with an anti‐fluorescence quencher, as determined using laser scanning confocal microscopy (LSM780; Zeiss).

### 2.8 Phagocytosis analysis

The primary peritoneal macrophages from *INOS^+/+^* or *INOS^−/−^* mice were isolated and cultured. After PA stimulation, phagocytic lipids were detected using Oil Red O staining. Real‐time polymerase chain reaction (PCR) was used to detect *CD36*, peroxisome proliferators‐activated receptors α (*PPARα*), perilipin 2 (i.e., *PLIN2*), ATP‐binding cassette transporter A1 (*ABCA1*), and carnitine palmitoyltransferase 1A (*CPT1A*) expression levels.

### 2.9 Quantitative real‐time PCR (qRT‐PCR)

Peritoneal macrophages from *INOS^+/+^* or *INOS^−/−^* mice were isolated and cultured, and the cells were stimulated with PA. Total RNA was extracted using TRIzol reagent (Invitrogen/Thermo Fisher Scientific) and used for complementary DNA synthesis with the PrimeScript^TM^ RT Reagent Kit with DNA Eraser (TaKaRa) following the manufacturer's instructions. qRT‐PCR for the genes of interest was performed using a LightCycler CFX96 (Bio‐Rad) under the conditions: preincubation at 95°C for 10 s, followed by 40 cycles of 95°C for 2 s, 55°C for 5 s, 72°C for 35 s, then 72°C for 5 min. Table 1 lists the specific primers.

**Table 1:** Quantitative real‐time polymerase chain reaction primers.

Gene name	Forward	Reverse
*INOS*	CTGCAGCACTTGGATCAGGAACCTG	GGAGTAGCCTGTGTGCACCTGGAA
*CD36*	ATGGGCTGTGATCGGAACTG	GTCTTCCCAATAAGCATGTCTCC
*PPARα*	TACTGCCGTTTTCACAAGTGC	AGGTCGTGTTCACAGGTAAGA
*PLIN2*	GACCTTGTGTCCTCCGCTTAT	CAACCGCAATTTGTGGCTC
*ABCA1*	GCTTGTTGGCCTCAGTTAAGG	GTAGCTCAGGCGTACAGAGAT
*TNF‐α*	CCCTCACACTCAGATCATCTTCT	GCTACGACGTGGGCTACAG
*IL‐6*	TAGTCCTTCCTACCCCAATTTCC	TTGGTCCTTAGCCACTCCTTC
*IL‐1β*	GCAACTGTTCCTGAACTCAACT	ATCTTTTGGGGTCCGTCAACT
*β‐actin*	GGCTGTATTCCCCTCCATCG	CCAGTTGGTAACAATGCCATGT

Abbreviations: *ABCA1*, ATP‐binding cassette transporter A1; *CD36*, cluster of differentiation 36; *IL6*, interleukin‐6; *IL‐1β*, interleukin‐1β; *INOS*, inducible nitric oxide synthase; *PLIN2*, perilipin 2; *PPARα*, peroxisome proliferators‐activated receptors α; *TNF‐α*, tumor necrosis factor‐*α*.

Each experiment was performed in triplicate. Transcript levels of the target genes were calculated by the $2^{‐∆∆C_{T}}$ method. All primers were synthesized by the Beijing Genomics Institution.

### 2.10 WB

Cells and liver tissues were harvested and lysed with radioimmunoprecipitation assay buffer, and then the protein concentrations were determined and quantified by bicinchoninic acid assay. Proteins were separated by 12% sodium dodecyl sulfate‐polyacrylamide gel electrophoresis and transferred to a polyvinylidene difluoride membrane (Millipore). After blocking with 5% BSA, the membranes were incubated with primary antibodies: anti‐INOS, anti‐LC3A/B, anti‐P62, anti‐mammalian target of rapamycin (mTOR), and phosphorylated‐mTOR (P‐mTOR) (1:1000 dilution; Cell Signaling Technology); anti‐β‐actin (1:1000 dilution; Proteintech); and anti‐GAPDH (1:5000 dilution; OriGene). After washing with Tris‐buffered saline with Tween 20, the membranes were incubated with peroxidase‐conjugated secondary antibodies (goat anti‐rabbit immunoglobulin G [IgG] or goat anti‐mouse IgG; Proteintech). Bound peroxidase activity was detected with an Enhanced Chemiluminescence Detection System (ECL, Thermo Fisher Scientific) using Immobilon^TM^ Western Chemiluminescent Horseradish Peroxidase Substrate (Merck Millipore).

### 2.11 Statistical analyses

Statistical differences between any two groups were evaluated using the independent sample double‐test *t* test, and the values were expressed as means ± standard deviations. Each experiment was performed at least three times. *p* values of <.05 were considered statistically significant. All statistical analyses were performed using GraphPad Prism 5.0 software (GraphPad Software).

## 3 RESULTS

### 3.1 NAFLD microenvironments enhance INOS expression in macrophages

**Figure 1:**
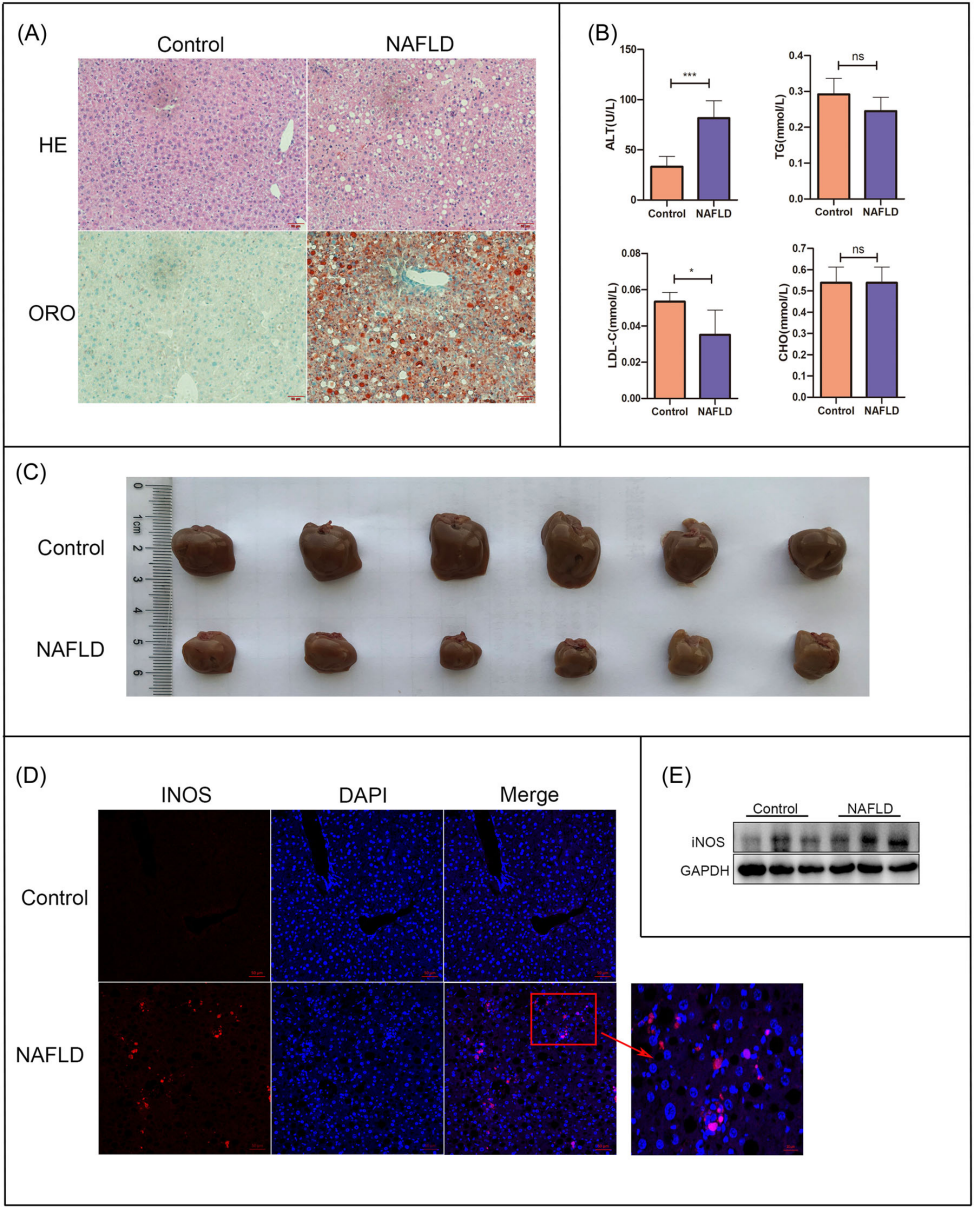
NAFLD models established by feeding wild‐type mice an MCD diet. (A) Immunohistochemical analyses of liver tissues from the NAFLD and control (ND) groups (*n* = 6 per group). Scale bar: 50 μm. (B) Serum ALT, TG, LDL‐C, and CHO levels. (C) Liver volume. (D, E) INOS expression based on (D) immunofluorescence (stained with DAPI [blue]; scale bar: 50 μm) and (E) western blot analysis. *p < .05 and ***p < .001 versus the control group. ALT, alanine aminotransferase; CHO, cholesterol; DAPI, 4′,6‐diamidino‐2‐phenylindole; GAPDH, glyceraldehyde 3‐phosphate dehydrogenase; HE, hematoxylin and eosin; INOS, inducible nitric oxide synthase; LDL‐C, low‐density lipoprotein cholesterol; MCD, methionine‐ and choline‐deficient; NAFLD, nonalcoholic fatty liver disease; ND, normal diet; ns, not significant; ORO, Oil Red O; TG, triglyceride.

The livers of the MCD diet model group were yellow and felt greasy compared to those of the control (normal diet) group. Furthermore, HE and Oil Red O staining indicated widespread steatosis. Increased serum ALT concentrations were also observed, indicating that the model was successfully created (Figure 1A–C). WB and immunofluorescence analyses also confirmed that INOS expression in the liver tissues was significantly higher in the NAFLD model group than in the control group (Figure 1D,E).

**Figure 2:**
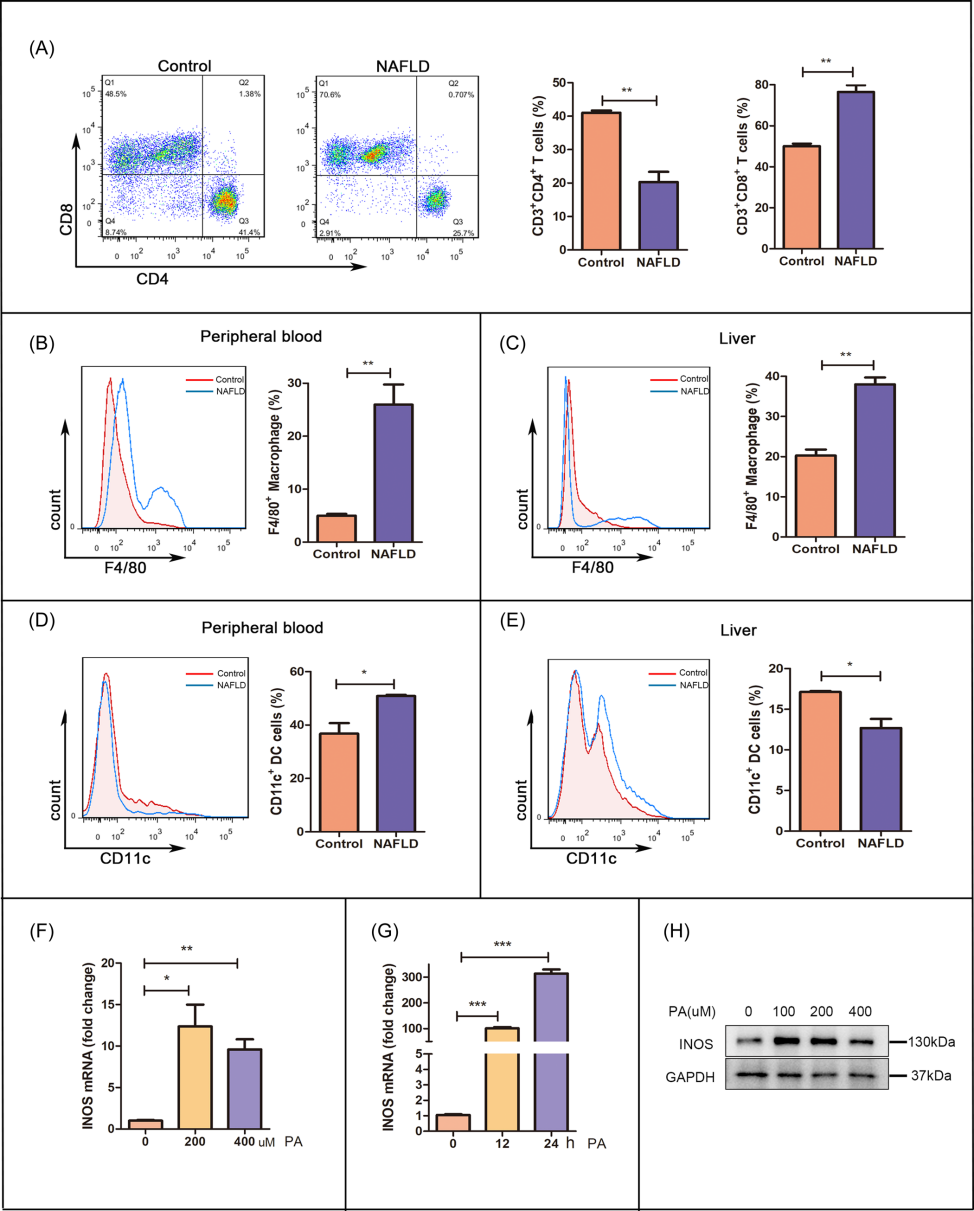
The NAFLD microenvironment enhances INOS expression in macrophages. (A) The percentage of CD3^+^CD4^+^ and CD3^+^CD8^+^ T cells from the peripheral blood of control and NAFLD mice (flow cytometry). (B–E) The numbers of F4/80^+^ macrophages and CD11c^+^ dendritic cells from the peripheral blood and liver of control and NALFD mice (flow cytometry). (F–H) INOS mRNA and protein levels in PA‐stimulated primary peritoneal macrophages. *p < .05, **p < .01, and ***p < .001 versus the control group. CD, cluster of differentiation; GAPDH, glyceraldehyde 3‐phosphate dehydrogenase; INOS, inducible nitric oxide synthase; mRNA, messenger RNA; NAFLD, nonalcoholic fatty liver disease; PA, palmitic acid.

To explore the changes in the liver's immune microenvironment during NAFLD progression, we evaluated the number of immune cells in the liver and peripheral blood, including dendritic cells, T cells, and macrophages. The flow cytometry results indicated that the number of immune cells significantly differed between the NAFLD and control groups, and macrophages differed the most (Figure 2A–E). We also confirmed enhanced INOS expression in the macrophages in these models. PA stimulation of macrophages in vitro (to mimic the NAFLD microenvironment) resulted in a significant and time‐dependent increase in INOS expression in the PA‐stimulated macrophages, confirmed by WB and qRT‐PCR (Figure 2F–H). These data strongly indicate that the NAFLD microenvironment enhanced INOS expression in macrophages.

### 3.2 *INOS* ^*−/−*^ alleviates steatosis with a HFD diet

**Figure 3:**
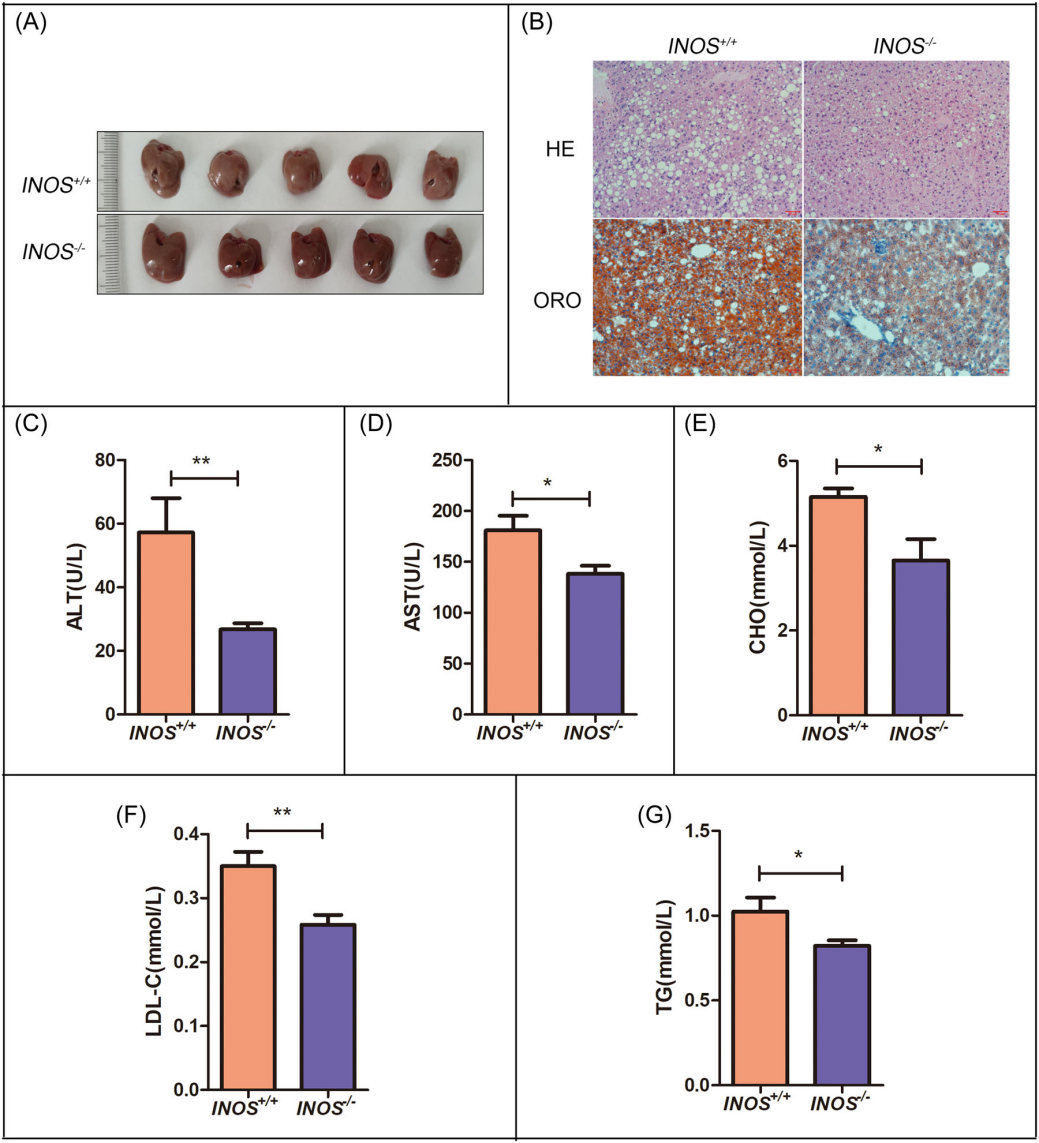
*INOS* ^−/−^ alleviates steatosis in mice with NAFLD induced by a HFD. (A) Liver volumes, (B) liver tissue HE and ORO staining (scale bar: 50 μm), and (C–G) serum ALT, AST, CHO, LDL‐C, and TG levels in *INOS* ^+/+^ (*n* = 5) and *INOS* ^−/−^ (*n* = 5) mice. *p < .05 and **p < .01 versus *INOS* ^+/+^ group. ALT, alanine aminotransferase; AST, aspartate aminotransferase; CHO, cholesterol; HE, hematoxylin and eosin; HFD, high‐fat diet; *INOS* ^−/−^, inducible nitric oxide synthase knockout mice; *INOS* ^+/+^, wild‐type mice; LDL‐C, low‐density lipoprotein cholesterol; NAFLD, nonalcoholic fatty liver disease; ORO, Oil Red O; TG, triglyceride.

To assess the effects of increased INOS expression in the NAFLD models, we developed an HFD diet‐induced NAFLD model in *INOS* ^+/+^ and *INOS* ^−/−^ mice that had been genotyped by PCR (Supporting Information S1: Figure 1 presents a representative genotype). After 4 months on an HFD, the *INOS* ^−/−^ mice exhibited markedly minor lipid accumulation (Figure 3A), palliative characteristic hepatic ballooning vacuolization in HE‐stained sections, less lipid deposition in Oil Red O‐stained sections (Figure 3B), and lower ALT, AST, CHO, TG, and LDL‐C concentrations (Figure 3C–G) compared to the controls, demonstrating that *INOS* knockout alleviated HFD‐induced liver injury.

**Figure 4:**
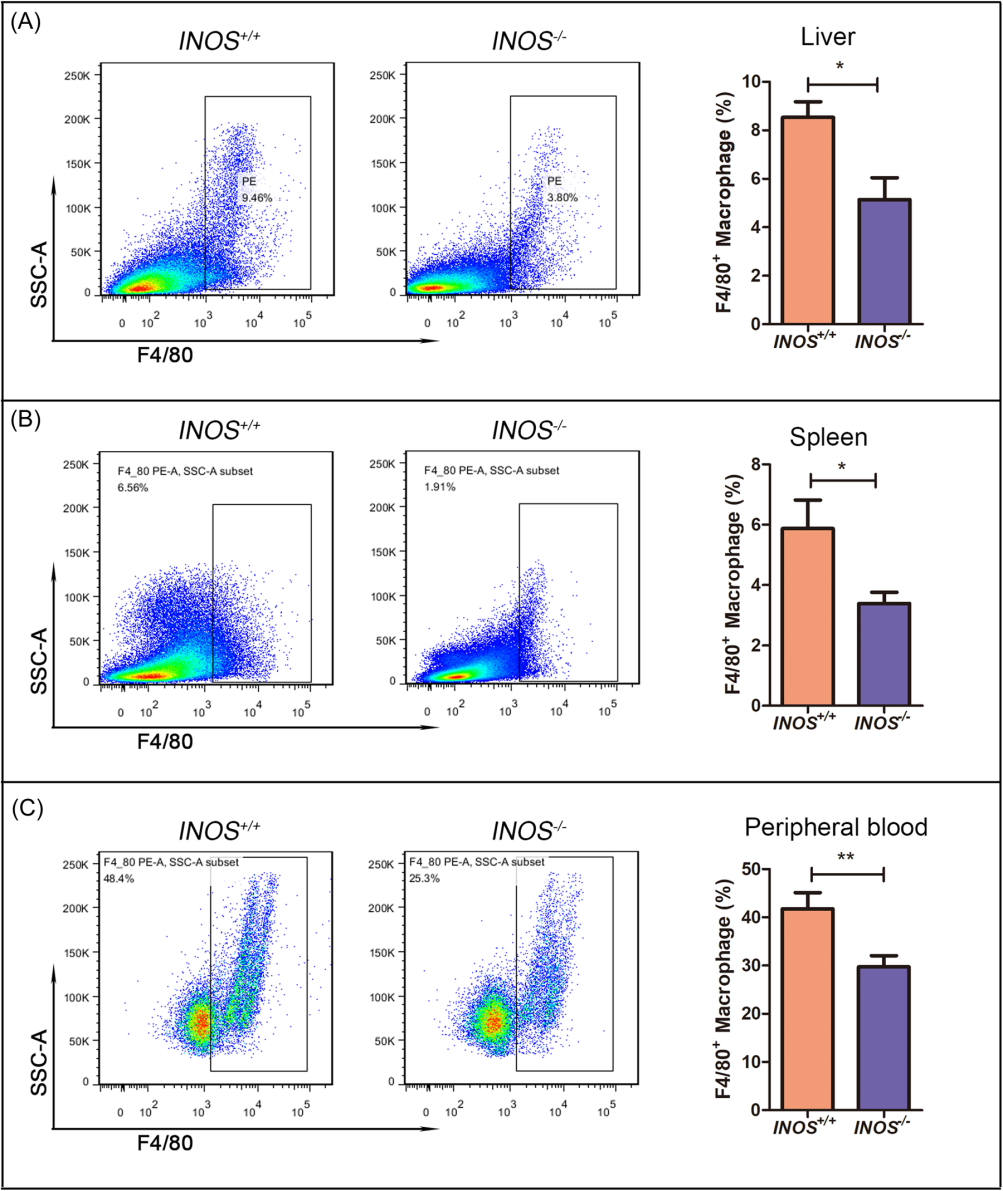
The number of macrophages is markedly lower in *INOS* ^−/−^ mice with NAFLD induced by a high‐fat diet than in *INOS* ^+/+^ mice. (A–C) The number of F4/80^+^ macrophages in the (A) liver, (B) spleen, and (C) peripheral blood of *INOS* ^+/+^ (*n* = 5) and *INOS* ^−/−^ (*n* = 5) mice (flow cytometry). *p < .05 and **p < .01 versus *INOS* ^+/+^ group. *INOS* ^−/−^, inducible nitric oxide synthase knockout mice; *INOS* ^+/+^, wild‐type mice.

Next, we performed a flow cytometry assay to further explore the relationship between improved NAFLD symptoms and macrophages, finding significant differences in the number of macrophages between the *INOS* ^−/−^ and *INOS* ^+/+^ mice; specifically, the *INOS* ^−/−^ group had significantly fewer macrophages than the *INOS* ^+/+^ group (Figure 4A–C). Previous studies have reported increased numbers of macrophages in the liver and peripheral blood under pathological conditions compared to the control groups, but *INOS* knockout reversed these effects and alleviated injury, suggesting a relationship between knockout‐*INOS* and remission.

### 3.3 *INOS* ^*−/−*^ reduces inflammatory cytokine production in macrophages

**Figure 5:**
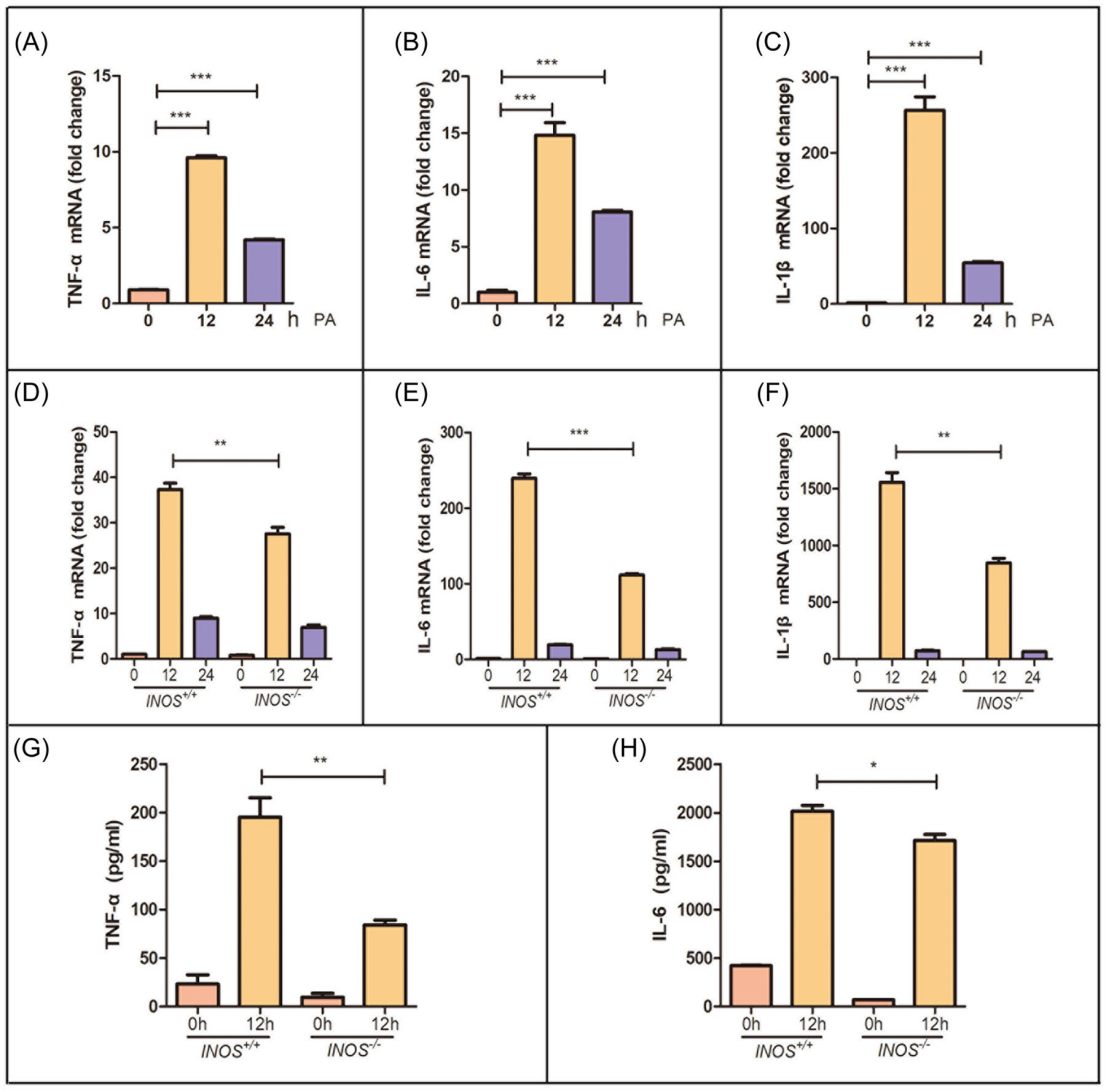
*INOS* ^−/−^ reduces inflammatory cytokine production in macrophages. (A–F) *TNF‐α*, *IL‐6*, and *IL‐1β* mRNA expression in primary peritoneal macrophages after PA stimulation for 12 and 24 h in (A–C) wild‐type and (D, E) *INOS* ^+/+^ and *INOS* ^−/−^ mice. (G, H) *TNF‐α*, *IL‐6* were detected in primary peritoneal macrophages by ELISA after PA stimulation for 12 in *INOS* ^+/+^ and *INOS* ^−/−^ mice. *INOS* ^−/−^, inducible nitric oxide synthase knockout mice; *INOS* ^+/+^, wild‐type mice; mRNA, messenger RNA; PA, palmitic acid; *TNF‐α*, tumor necrosis factor‐α. *p < .05, **p < .01, and ***p < .001.

Next, to elucidate the role of INOS in macrophage regulation, we stimulated peritoneal macrophages from *INOS* ^+/+^ and *INOS* ^−/−^ mice with PA and measured inflammatory cytokine expression. The interleukin (*IL*)‐*1β*, *IL‐6*, and *TNF‐α* messenger RNA levels in the macrophages increased significantly following PA stimulation, but the inflammatory cytokines levels dramatically decreased in *INOS* ^−/−^ mice compared to the *INOS* ^+/+^ mice, emphasizing an association between INOS and macrophage immune function (Figure 5).

### 3.4 *INOS* ^−/−^ mice have fewer M1 macrophages

In vitro, *INOS* knockout mitigated the increased levels of inflammatory factors after PA stimulation. Thus, we used an NAFLD disease model to further explore the effects of INOS on macrophages, finding that the number of M1 macrophages decreased significantly after *INOS* knockout in the liver, but the number of M2 macrophages did not differ between the knockout and control mice (Supporting Information S1: Figure 2).

Previous studies reported that M1 macrophages promote inflammation in NAFLD, and INOS regulates M1 macrophage growth and development. Thus, we detected the types of macrophages in the spleen and peripheral blood, finding that the number of M1 macrophages decreased significantly after *INOS* knockout (Supporting Information S1: Figures 3 and 4). Therefore, decreased levels of inflammatory factors after *INOS* knockout in NAFLD might be due to decreased levels of M1 macrophages resulting from *INOS* knockdown.

### 3.5 Macrophages from *INOS* ^*−/−*^ mice have enhanced phagocytic ability

**Figure 6:**
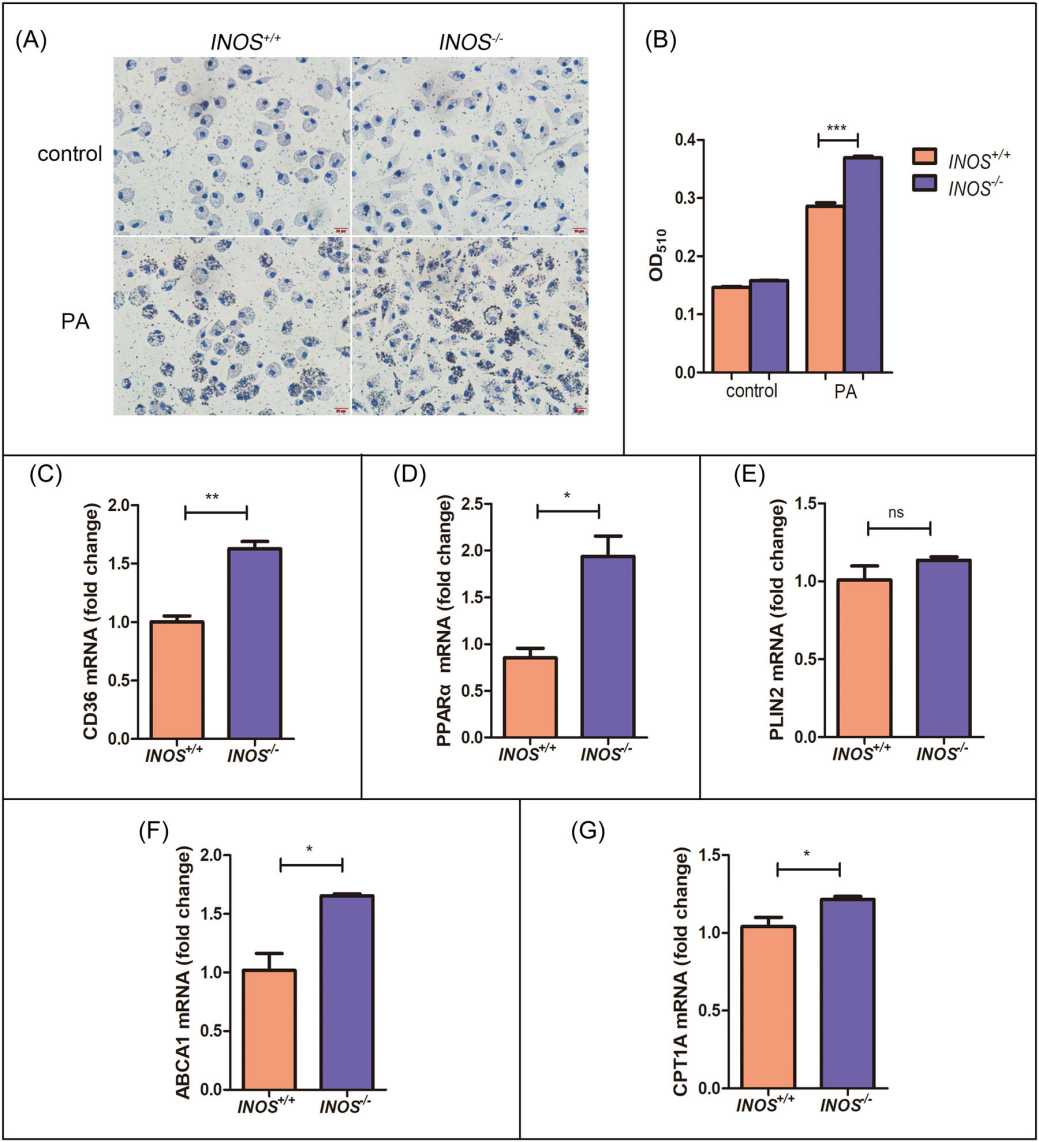
*INOS^−/−^* macrophages have enhanced phagocytic abilities. (A) Oil Red O staining (scale bar: 20 μm) and (B) quantification of primary peritoneal macrophages obtained from *INOS* ^+/+^ and *INOS^−/−^* mice with and without PA stimulation. (C–G) *CD36*, *PPARα*, *PLIN2*, *ABCA1*, and *CPT1A* mRNA expression in PA‐stimulated primary peritoneal macrophages obtained from *INOS* ^+/+^ and *INOS^−/−^* mice. *ABCA1*, ATP‐binding cassette transporter A1; *CD36*, cluster of differentiation 36; *CPT1A*, carnitine palmitoyltransferase 1A; *INOS^−/−^*, inducible nitric oxide synthase knockout mice; *INOS* ^+/+^, wild‐type mice; mRNA, messenger RNA; ns; not significant; PA, palmitic acid; *PLIN2*, perilipin 2; *PPARα*, peroxisome proliferators‐activated receptors α. *p < .05, **p < .01, and ***p < .001.

Under pathological conditions, monocytes recruited from the blood enter the liver and phagocytose to slow disease progression. In this study, we observed significantly more Oil Red O‐positive cells in *INOS* ^−/−^ macrophages than in *INOS* ^+/+^ macrophages, especially after PA stimulation (Figure 6A,B). To explain this phenomenon, we measured the expression levels of phagocytosis‐related receptors, finding markedly upregulated *CD36* and *PPARα* expression in *INOS* ^−/−^ macrophages (Figure 6C,D). Receptors expressed on monocytes/macrophages are associated with lipoprotein uptake, and PPARα plays a crucial role in lipid metabolism (e.g., lipogenesis, lipolysis, lipid transport, and oxidation), amino acid metabolism, gluconeogenesis, and ketogenesis; thus, it has considerable potential as a treatment target for obesity and diabetes. Furthermore, *ABCA1* (a receptor related to reverse CHO transport) and *CPT1A* expressions were significantly higher in *INOS* ^−/−^ macrophages than in *INOS* ^+/+^ macrophages (Figure 6F,G). Increased *CPT1A* expression improves biological particle uptake, suggesting that INOS participates in NAFLD by regulating lipid homeostasis.

### 3.6 INOS expression increases in hepatic macrophages and correlates with autophagy

**Figure 7:**
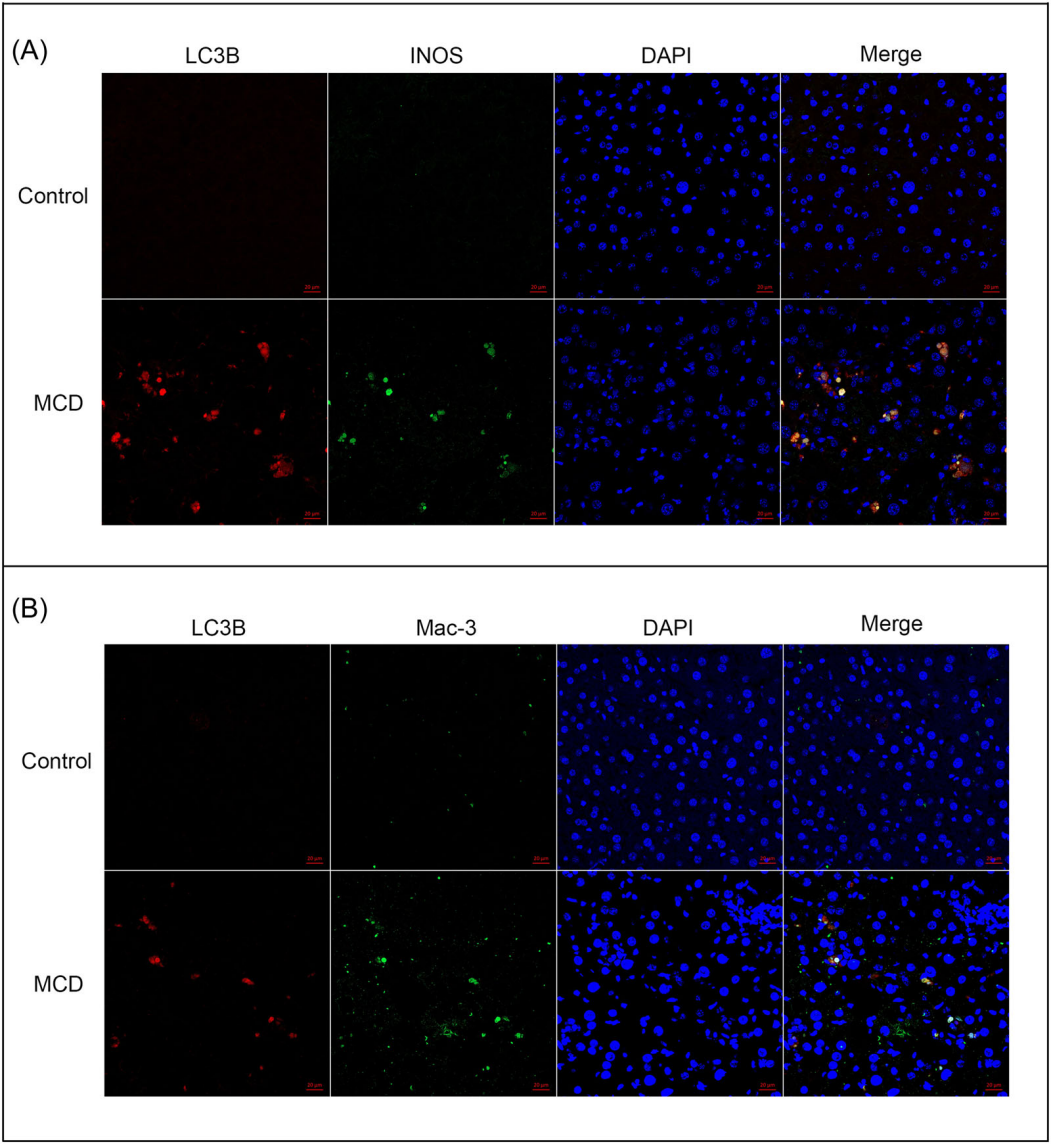
INOS expression increases in hepatic macrophages from mice with MCD‐induced NAFLD and correlates with autophagy. Immunofluorescence staining of liver tissue from wild‐type and NAFLD mice for co‐expressing (A) INOS and LC3B and (B) Mac‐3 and LC3B. Scale bar: 20 μm. DAPI, 4′,6‐diamidino‐2‐phenylindole; INOS, inducible nitric oxide synthase; LC3B, light chain 3B; MCD, methionine‐ and choline‐deficient; NAFLD, nonalcoholic fatty liver disease.

To explore the relationship between INOS expression in the liver and macrophage autophagy in NAFLD, we used an MCD diet to generate an NAFLD mouse model and then collected liver tissues for immunofluorescence staining. INOS expression was significantly higher in the NAFLD model group than in the control group (Figure 7A). Furthermore, LC3B expression was detected in the liver, suggesting a correlation between INOS and LC3B (Figure 7A) and that INOS might be involved in autophagy progression. In addition, the autophagy levels of macrophages changed significantly under pathological conditions (Figure 7B). Therefore, INOS may participate in and regulate the autophagy level of macrophages, contributing to disease progression.

### 3.7 *INOS* ^−/−^ macrophages have enhanced autophagy flux

**Figure 8:**
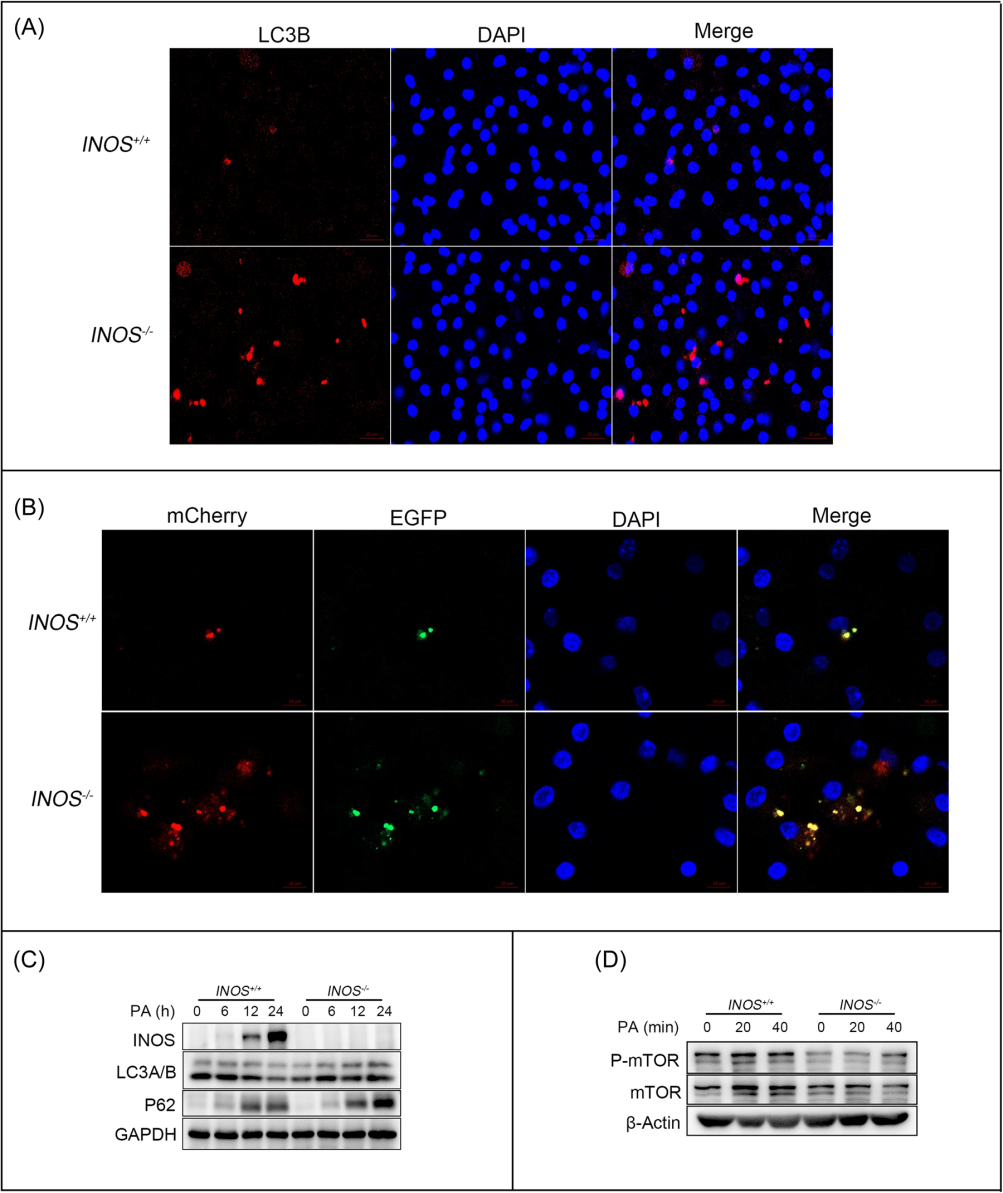
Macrophages from INOS^−/−^ mice have enhanced autophagy flux. (A) LC3B expression in primary peritoneal macrophages from *INOS* ^+/+^ and *INOS* ^−/−^ mice. Scale bar: 20 μm. (B) Autophagy flux of macrophages isolated from *INOS* ^+/+^ and *INOS* ^−/−^ and visualized via mCherry‐EGFP‐LC3 adenovirus. Scale bar: 10 μm. (C, D) Protein expression of INOS, LC3A/B, P62, mTOR, and P‐mTOR protein expression in PA‐stimulated macrophages isolated from *INOS* ^+/+^ and *INOS* ^−/−^ mice. DAPI, 4′,6‐diamidino‐2‐phenylindole; EGFP, enhanced green fluorescent protein; GAPDH, glyceraldehyde 3‐phosphate dehydrogenase; INOS, inducible nitric oxide synthase; *INOS* ^−/−^, inducible nitric oxide synthase knockout mice; *INOS* ^+/+^, wild‐type mice; LC3B, light chain 3B; mTOR, mammalian target of rapamycin; PA, palmitic acid; P‐mTOR, phosphorylated‐mTOR.

Primary peritoneal macrophages were isolated from *INOS* ^−/−^ and *INOS* ^+/+^ mice, then stimulated with PA to further explore the effects of INOS on macrophage autophagy. Immunofluorescence staining demonstrated that LC3B expression was significantly higher in primary peritoneal macrophages derived from *INOS* ^−/−^ mice than from *INOS* ^+/+^ mice (Figure 8A). We also detected autophagy flow through the mCherry‐EGFP‐LC3 adenovirus to confirm the regulatory effects of INOS on autophagy flux, finding enhanced autophagy after *INOS* knockout (Figure 8B). Furthermore, based on P62 and LC3A/B protein expression, INOS expression increased and LC3A/B expression decreased as the PA stimulation time increased (Figure 8C). However, after *INOS* knockout, the autophagy level significantly increased with PA stimulation (Figure 8C), suggesting that INOS is involved in macrophage autophagy under PA stimulation.

To elucidate how INOS regulates macrophage autophagy, we measured the levels of key mTOR signaling pathway molecules after PA treatment of primary peritoneal macrophages from *INOS^−/−^* and *INOS^+/+^* mice. P‐mTOR decreased significantly after *INOS* knockdown (Figure 8D), demonstrating that INOS affects autophagy in macrophages through the mTOR signaling pathway.

## 4 DISCUSSION

Dietary structures have changed with rapid lifestyle and social economy changes, which has increased the incidence of NAFLD annually. Consequently, NAFLD has become the main cause of chronic liver disease worldwide and seriously threatens public health. Prospective studies suggest that NAFLD will become the most important cause of end‐stage liver disease and liver transplantation in the next decade, severely burdening the national social economy.^1^ The specific mechanisms of NAFLD development have not been fully elucidated, but our study found significantly increased INOS levels in an NAFLD model. INOS is closely related to the immune response, oxidative damage, and the inflammatory response.^23^ Notably, INOS is mainly expressed in macrophages and is inextricably linked to NAFLD progression, particularly those in the liver tissue, including aboriginal Kupffer cells and monocytes from the blood circulation.^22^ Our study found that the number of macrophages in the peripheral blood and liver of mice with NAFLD increased significantly. Furthermore, in vitro macrophage stimulation by PA increased INOS expression. These results suggest that INOS helps regulate NAFLD development and progression.

We constructed an NAFLD model in mice with and without *INOS*, finding that *INOS* knockout slowed NAFLD progression but decreased the number of macrophages in the peripheral blood and liver. Studies have reported that INOS regulates macrophage production^24^; therefore, it is plausible that the loss of INOS reduces macrophage production. We also confirmed that macrophages without INOS had significantly lower levels of related inflammatory factors stimulated by PA. The phagocytic function of the macrophages was also explored, finding that *INOS* knockdown regulated *CD36*, *PPARα*, and *ABCA1* expression in macrophages. CD36 and PPARα in macrophages participate in lipid uptake. Moreover, ABCA1 dysfunction promotes large CHO deposits in macrophages, forming foam cells, which affects macrophage‐related functions; we found upregulated *ABCA1* expression after *INOS* knockout. Moreover, increased *CPT1A* expression restores macrophage phagocytosis, and *CPT1A* knockdown in macrophages harms macrophage phagocytosis. Our study found increased CPT1A levels after *INOS* knockout, confirming our hypothesis that INOS participates in and regulates the macrophage phagocytosis of lipids. In this process, INOS promotes macrophage lipid phagocytosis and CHO transport to promote the treatment of lipids by macrophages and slow the fatty liver process.

**Figure 9:**
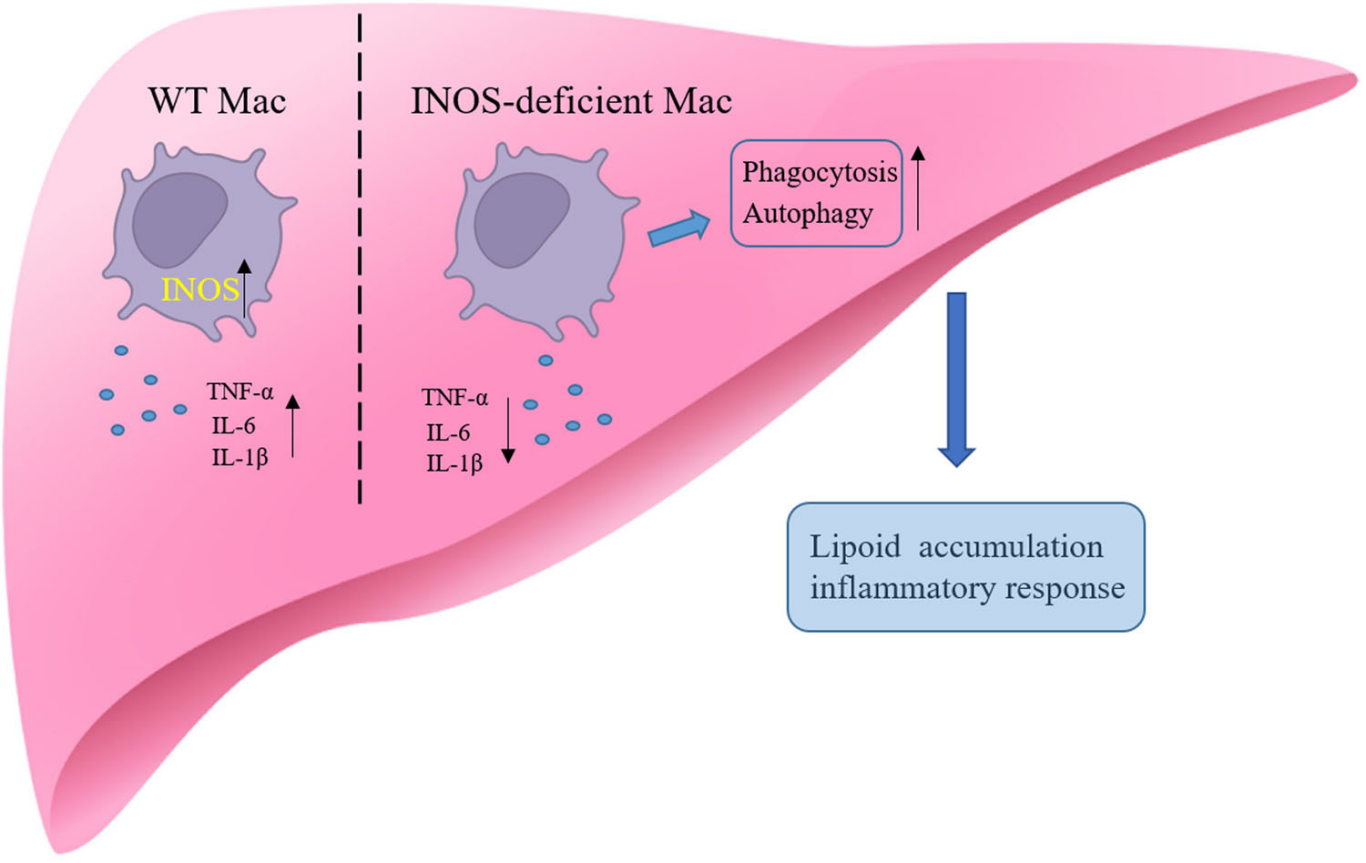
Macrophage autophagy level increased and decreased levels of inflammatory cytokines in the absence of INOS, promoting the metabolism of macrophages to dead cells and lipids and slowing NAFLD progression. *IL‐1β*, interleukin‐1β; *IL‐6*, interleukin‐6; *INOS*, inducible nitric oxide synthase; *Mac*, macrophage; *TNF‐α*, tumor necrosis factor‐*α*; *WT*, wild‐type.

Autophagy is a “self‐eating” phenomenon common in most eukaryotic cells, which is the phagocytic degradation and recycling mechanism of lysosomes to its own structure. Autophagy eliminates cells' metabolic waste via autophagy bodies and lysosome binding. Autophagy is also a protective mechanism as it helps maintain homeostasis and self‐regulation in response to stress. Under certain stress conditions, the autophagy of cells is enhanced, which helps prevent or slow the progression of some diseases.^25^ Autophagy is strongly associated with the pathogenesis of NAFLD, including cellular glucose and lipid metabolism regulation, insulin sensitivity, mediating hepatocyte resistance to damaging stimuli, and preventing overactivation of the innate immune response, which are crucial for the occurrence and development of NAFLD.^26^ Autophagy is defective in NAFLD liver tissues, and in patients with NASH and HFD diet‐induced NAFLD mouse models, the autophagy level in liver macrophages decreases, promoting the phagocytosis of apoptotic hepatocytes by macrophages. Therefore, targeting macrophage autophagy might be a new strategy for treating NAFLD metabolism and inflammation‐related diseases. Studies have demonstrated that autophagy regulates INOS expression and inflammation levels. However, none have demonstrated whether INOS regulates autophagy. Our study found that the macrophage autophagy level increased in the absence of INOS, promoting the metabolism of macrophages to dead cells and lipids and slowing NAFLD progression (Figure 9).

This study has certain limitations. Liver macrophages are known as Kupffer cells. Thus, to better understand the function of macrophages in the liver, Kupffer cells should be selected for further studies. In addition, the correlation between INOS and autophagy was not verified in the animal models and requires further exploration.

## 5 CONCLUSION

This study confirms that INOS is involved in NAFLD progression by influencing macrophage‐mediated changes in lipid metabolism and autophagy, providing a new research basis for treating NAFLD.

## AUTHOR CONTRIBUTIONS

*Conceptualization*: Guiyuan Jin and Xiaoying Yao. *Data curation*: Guiyuan Jin, Dong Liu, Juan Zhang, Yonghong Yang, Yanzhen Bi, Hui Zhang, Guanjun Dong, and Shumin Cheng. *Formal analysis*: Guiyuan Jin and Huixin Tang. *Funding acquisition*: Feng Hong, Meng Si, and Guiyuan Jin. *Writing*: Guiyuan Jin and Meng Si.

## CONFLICT OF INTEREST STATEMENT

The authors declare no conflict of interest.

## ETHICS STATEMENT

The Affiliated Hospital of Jining Medical College approved the animal ethics permit for this study (2021‐08‐C018).

## Supporting information

Supporting information.

## Data Availability

Share research data including: raw data, software, protocols, methods, and material.
