# Supplementary material for: Inducible nitric oxide synthase accelerates nonalcoholic fatty liver disease progression by regulating macrophage autophagy
Source: Immun Inflamm Dis. 2023 Dec 26;11(12):e1114. doi: 10.1002/iid3.1114 (PMC10750437; doi:10.1002/iid3.1114)
Supplement: Supplementary file 1 — Supporting information. [file IID3-11-e1114-s001.docx]

Supplemental Fig. 1


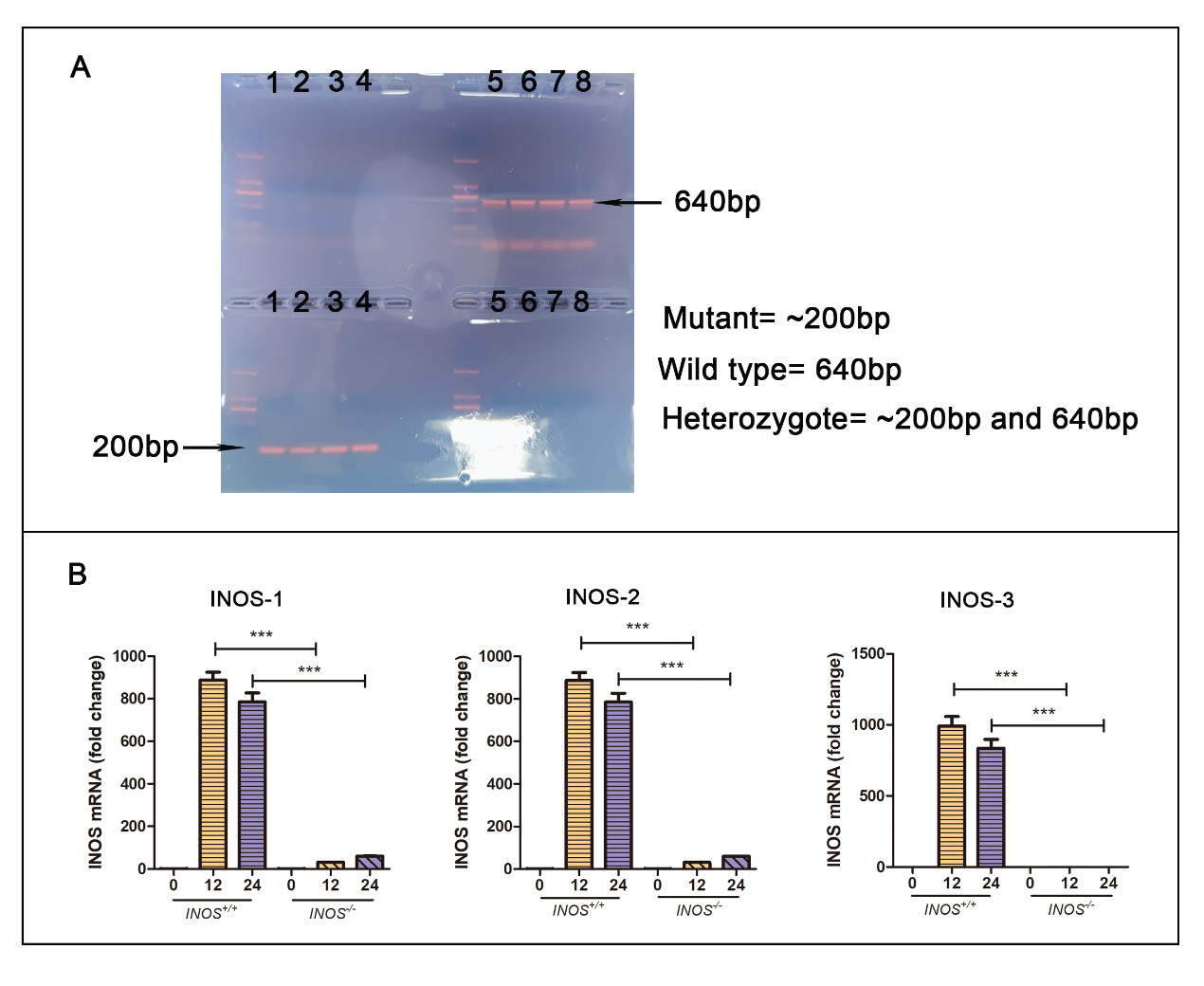


Supplemental Figure 1. Validation of the *INOS^-/-^* mouse model.

(A) *INOS^-/-^* mice were confirmed by agarose gel electrophoresis. (B) *INOS-1*, *2*, and *3* mRNA expression in macrophages following PA stimulation.

***P <0.001.

INOS: Inducible nitric oxide synthase; *INOS^-/-^*: Inducible nitric oxide synthase knockout mice.

Supplemental Fig. 2


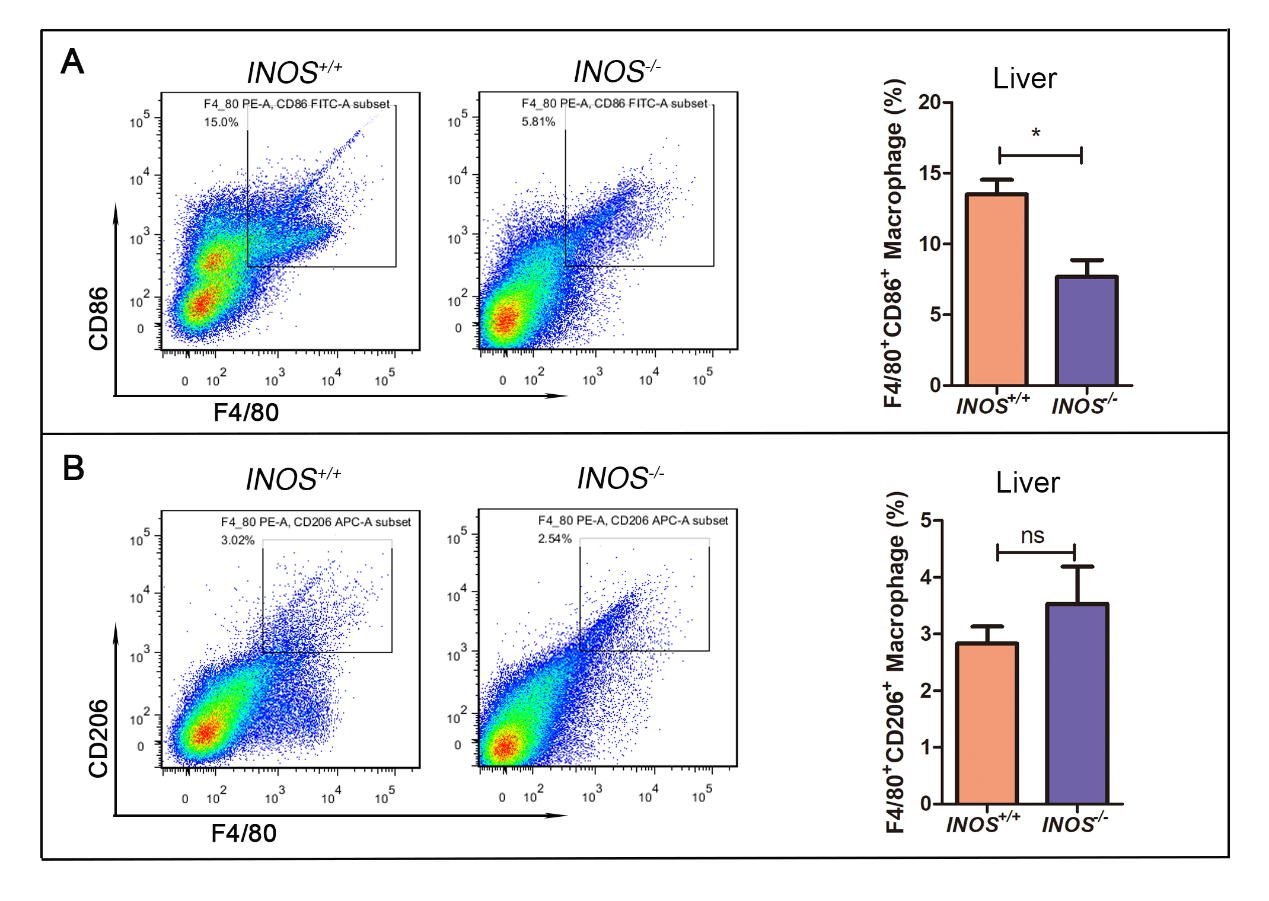


Supplemental Figure 2. The livers of high-fat diet-induced NAFLD *INOS^-/-^* mice have fewer M1 macrophages than *INOS^+/+^* mice.

(A, B) The gating strategies and percentages of (A) F4/80^+^CD86^+^ M1 and (B) F4/80^+^CD206^+^ M2 macrophages.

*P <0.05.

CD: Cluster of differentiation; *INOS^+/+^*: Mice with inducible nitric oxide synthase; *INOS^-/-^*: Inducible nitric oxide synthase knockout mice; ns: Not significant.

Supplemental Fig. 3


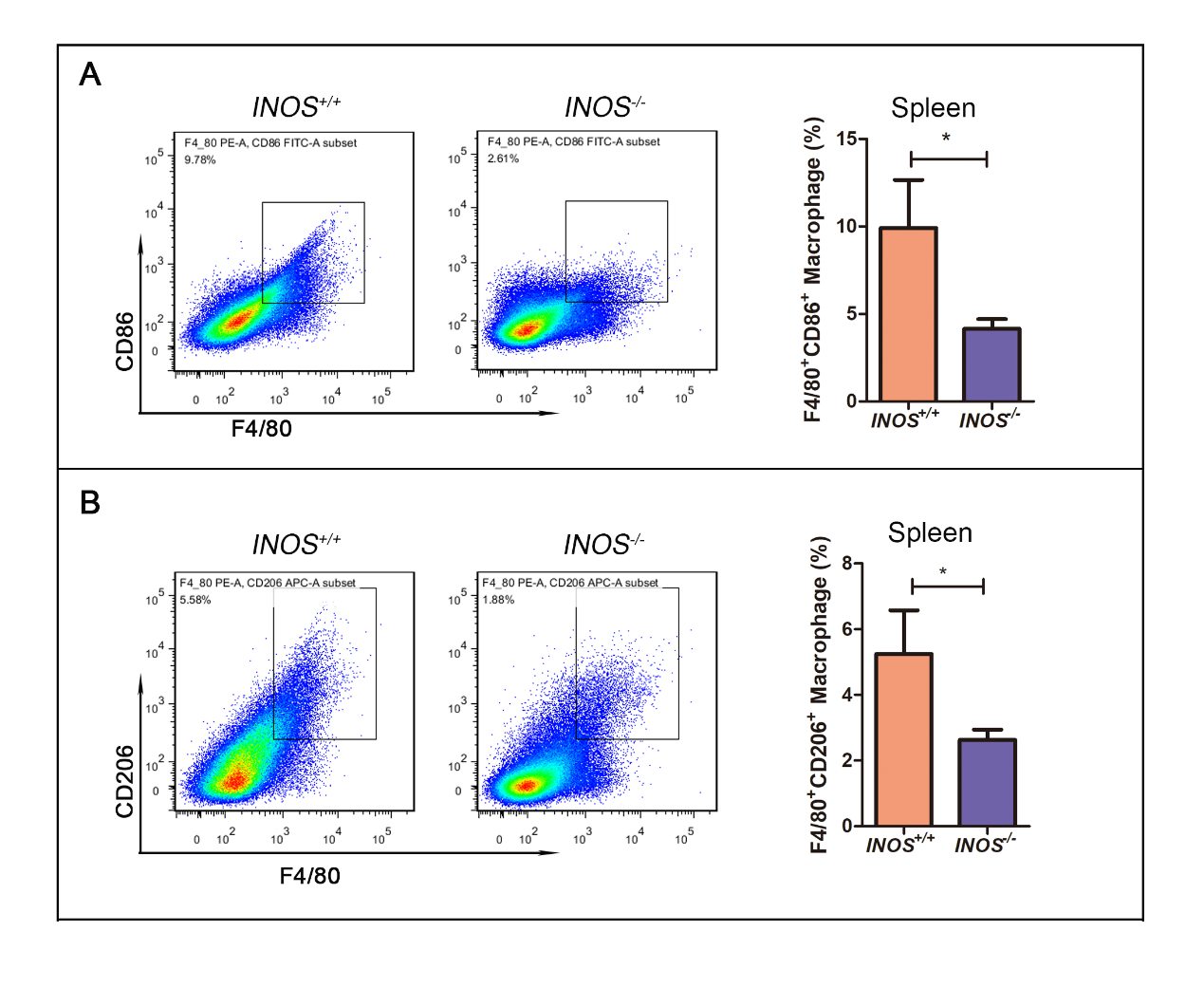


Supplemental Figure 3. The spleens of high-fat diet-induced NAFLD *INOS^-/-^* mice have fewer M1 macrophages than *INOS^+/+^* mice.

(A, B) The gating strategies and percentages of (A) F4/80^+^CD86^+^ M1 and (B) F4/80^+^CD206^+^ M2 macrophages.

*P <0.05.

CD: Cluster of differentiation; *INOS^+/+^*: Mice with inducible nitric oxide synthase; *INOS^-/-^*: Inducible nitric oxide synthase knockout mice.

Supplemental Fig. 4


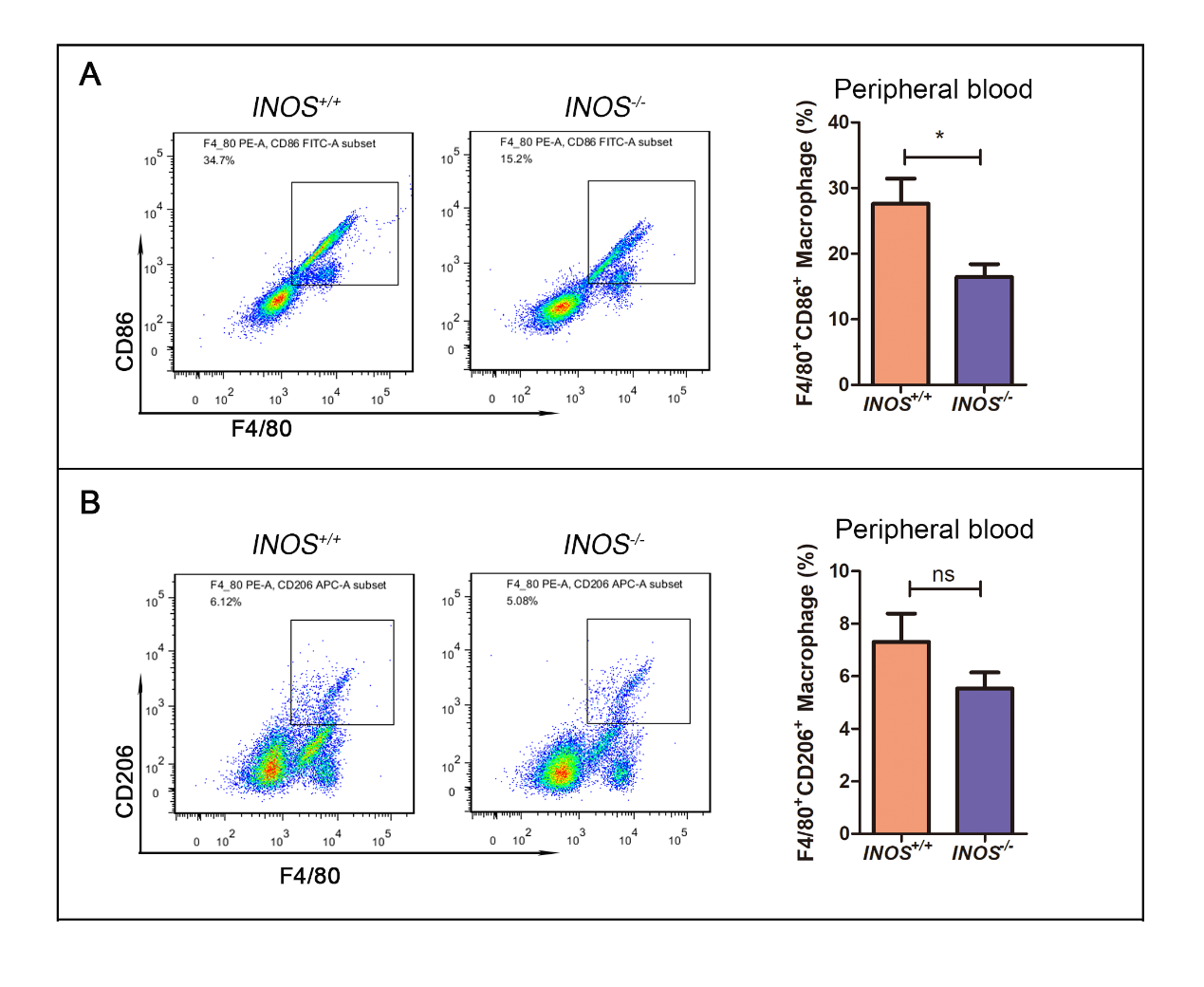


Supplemental Figure 4. The peripheral blood of high-fat diet-induced NAFLD *INOS^-/-^* mice has fewer M1 macrophages than *INOS^+/+^* mice.

(A, B) The gating strategies and percentages of (A) F4/80^+^CD86^+^ M1 and (B) F4/80^+^CD206^+^ M2 macrophages.

*P <0.05.

CD: Cluster of differentiation; *INOS^+/+^*: Mice with inducible nitric oxide synthase; *INOS^-/-^*: Inducible nitric oxide synthase knockout mice; ns: Not significant.
